# Towards Lightweight Neural Networks for Garbage Object Detection

**DOI:** 10.3390/s22197455

**Published:** 2022-09-30

**Authors:** Xinchen Cai, Feng Shuang, Xiangming Sun, Yanhui Duan, Guanyuan Cheng

**Affiliations:** 1 School of Electrical Engineering, Guangxi University, Nanning 530004, China; 2 College of Physical Science and Technology, Central China Normal University, Wuhan 430079, China

**Keywords:** garbage classification, object detection, dilated–deformable convolution, lightweight neural network

## Abstract

In recent years, garbage classification has become a hot topic in China, and legislation on garbage classification has been proposed. Proper garbage classification and improving the recycling rate of garbage can protect the environment and save resources. In order to effectively achieve garbage classification, a lightweight garbage object detection model based on deep learning techniques was designed and developed in this study, which can locate and classify garbage objects in real-time using embedded devices. Focusing on the problems of low accuracy and poor real-time performances in garbage classification, we proposed a lightweight garbage object detection model, YOLOG (YOLO for garbage detection), which is based on accurate local receptive field dilation and can run on embedded devices at high speed and with high performance. YOLOG improves on YOLOv4 in three key ways, including the design of DCSPResNet with accurate local receptive field expansion based on dilated–deformable convolution, network structure simplification, and the use of new activation functions. We collected the domestic garbage image dataset, then trained and tested the model on it. Finally, in order to compare the performance difference between YOLOG and existing state-of-the-art algorithms, we conducted comparison experiments using a uniform data set training model. The experimental results showed that YOLOG achieved $AP_{0.5}$ of 94.58% and computation of 6.05 Gflops, thus outperformed YOLOv3, YOLOv4, YOLOv4-Tiny, and YOLOv5s in terms of comprehensive performance indicators. The network proposed in this paper can detect domestic garbage accurately and rapidly, provide a foundation for future academic research and engineering applications.

## 1. Introduction

Garbage has become an unavoidable topic in modern society. Since the birth of humanity, garbage has been produced. Since the turn of the 21st century, productivity has increased rapidly, which has also caused the amount of garbage to proliferate. On average, each person in China generates 300 kg of domestic garbage annually. The amount of domestic garbage generated in China is about 400 million tons annually, growing at a rate of 8% per year [1]. Domestic garbage poses a significant threat to the sustainability of cities, especially in developing countries. It has become a primary source of environmental pollution in many cities. Precise and rational classification of waste and proper disposal of garbage can make efficient use of resources and reduce pollution. Therefore, the detection and classification of garbage are of great importance for sustainable urban development.

The Environmental Protection Agency (EPA) proposes municipal solid waste recycling as an effective strategy [2]. In fact, many cities are already exploring such strategies, such as Berlin and Singapore [3], which can make efficient use of resources and reduce pollution. On 31 January 2019, the Shanghai Municipal Regulations on Domestic Garbage Management [4] was officially implemented. As China’s first pilot city, Shanghai started implementing mandatory garbage classification. All domestic garbage is divided into four categories: recyclable, harmful, wet, and dry. Each category contains multiple types of garbage; hence, residents often have difficulties in identifying and remembering when sorting garbage, requiring the use of intelligent devices to assist in detecting and sorting. In addition, the recycling company needs to further sort the garbage after recycling, also requiring intelligent equipment to assist in identifying and sorting. A large amount of unsorted domestic garbage creates a significant workload for the staff and long-term work in this environment can be harmful to their health. The speed of manual garbage classification is too slow to solve the current problem of excessive domestic garbage production, and leads to garbage accumulation. Therefore, a system is urgently needed to help people in detecting and classifying garbage. A standard, uniform, fast, and efficient waste detection and sorting system is a vital tool to improve the efficiency of waste classification.

The development of deep learning techniques has provided new solutions for garbage classification. Many scholars have designed garbage classification algorithms based on deep learning techniques, such as garbage classification based on image classification techniques [5,6,7] and based on object detection techniques [8]. These algorithms can be applied to intelligent garbage sorting equipment to improve the efficiency of garbage sorting, such as intelligent trash cans, garbage sorters, intelligent garbage stations, and mobile apps. Garbage is usually transported on a conveyor belt, and the garbage needs to be detected and classified during the transmission process. In this process, it is necessary to ensure the real-time nature of the garbage recognition; otherwise, the controller will experience significant delays, resulting in classification failure. It is impractical to have a GPU on each device to achieve the requirement of real-time operations, which would lead to high cost and power consumption. Therefore, such a network needs to be designed to be as lightweight as possible while maintaining accuracy. Currently, significant progress has been made in lightweight deep learning models that can be smaller while ensuring accuracy, providing theoretical and technical support for implementing waste classification in devices with limited computing power

In order to solve the problem of a large number of model parameters and computation, many new convolution methods have been proposed, such as dilated convolution [9], deformable convolution [10], group convolution [11], and depth-wise separable convolution [12]. Many essential lightweight networks have been designed based on these new convolutional method designs. For example, MobileNetV2 [13] combines depth-wise separable convolution and the residual structure in ResNet [14] to reach 74.7% of Top 1 in the ImageNet image classification task with only 3.4 M parameters and 300 M computations; ShuffleNetV2 uses pointwise group convolutions, bottleneck-like structures, and channel split to outperform MobileNetV2 in classification and detection tasks regarding accuracy and speed [15]. Yang and Li designed a lightweight garbage classification model, WasNet, using depth-wise separable convolution combined with an attention mechanism, and achieved an accuracy of 82.5% with only 1.5 M parameters on a self-built data set [16]. Chen et al. designed a lightweight garbage classification model, GCNet, based on ShuffleNetV2, introducing a parallel mixed attention mechanism module (PMAM) and transfer learning, which presented 97.9% accuracy and 1.3 M parameters on a self-built data set [17].

In the above research, lightweight garbage classification is almost always treated as a classification task. However, classification tasks can only classify one object at a time. When there are multiple objects in the image or the background is complex, the classification task does not work well, and often cannot give the specific location of the garbage. The detection task can make up for these shortcomings. Garbage detection tasks have been rarely addressed in the academic literature, and even less research has been done on lightweight detection models in this area. Mao et al. used the YOLOv3 model for garbage object detection, with an $AP_{0.5}$ of 92.12% on the TRWD data set and a detection speed of 80 FPS using an RTX 2080 SUPER GPU [18]. Xi and Zhai combined SSD and YOLO to design a garbage object detection network with an AP of 69.87% on domestic garbage pictures in the “Huawei cloud Cup” 2020 Shenzhen Open Data Application Innovation Competition [19] and achieved 48 FPS on an Nvidia Tesla K20 GPU [20]. The above studies considered the use of GPU platforms, and it is impractical to equip each device with a GPU in practical applications. Therefore, in this work, we utilize the speed advantage of a lightweight neural network and the localization advantage of object detection for garbage classification, and design a lightweight real-time garbage object detection algorithm based on YOLOv4, which is a fixed-point quantized and can be applied in embedded devices.

The main contributions of this paper include the following.

- The proposed dilated–deformable convolution combines dilated convolution and deformable convolution to precisely dilate the local receptive field without increasing the number of parameters and computation.
- We optimized the network structure on the basis of YOLOv4 (to ensure accuracy) and significantly reduced the number of parameters and computation.

## 2. Related Work

Convolutional neural networks (CNNs) have been applied for image recognition in a large number of applications. In convolutional neural networks, garbage classification can be divided into classification and detection tasks. There has been much more research related to the classification task for garbage classification. Such models are very mature, the classification accuracy is high, and the models are lightweight. Related models include CompostNet [5], X-DenseNet [6], and WasNet [16]. However, the classification task can only identify one type of object in an image and is greatly affected by the environment. In contrast, the detection task can identify and locate multiple objects. Data augmentation during training can enrich the environment and objects, reducing the impact of the environment and multiple objects on the recognition results. There have been much less research studies related to garbage detection tasks compared to classification tasks, and even less on ensuring that the relevant model is lightweight. De Carolis et al. [8] and Mao et al. [18] used an improved YOLOv3 network model for garbage detection and recognition. However, they did not improve and reduce the weight of the network, retaining extensive computation. Xi and Zhai combined SSD and YOLO to design a garbage object detection network, which operates at 48 FPS on an Nvidia Tesla K20 GPU [20]. Qin et al. segmented garbage images, fused the segmented garbage images with complex backgrounds from other data sets, and trained YOLOv3 and Faster R-CNN models using the fused data sets to improve accuracy [21]. The above studies were designed based on the application of GPU devices, and the number of network parameters and computational burdens are extensive. Therefore, in this paper, we researched lightweight models for the garbage detection task, such that the model can operate at a high speed while maintaining the accuracy of edge devices. The following is a brief introduction to the current mainstream object detection networks and lightweight network methods, as well as a brief description of this research’s network selection and lightweight method.

CNN-based object detection models can mainly be divided into one- and two-stage detectors. One-stage detectors can directly obtain the final detection result through a single detection, while two-stage detectors divide the detection into two steps, first finding the candidate region and then classifying the candidate region. Representative one-stage detection models include YOLO, SSD, CenterNet, YOLOv3, YOLOv4, YOLOv5, and EfficientDet; while representative two-stage detectors are R-CNN, Fast R-CNN, Faster R-CNN, and Mask R-CNN. Zaidi et al. have tested state-of-the-art representative object detection networks, and experimentally showed that the inference speed of two-stage detectors is significantly slower than that of one-stage detectors, such that they cannot achieve real-time processing. Among the one-stage detectors, YOLO, YOLOv3, EfficientDet-D2, and YOLOv4 can ensure both real-time and high accuracy operations, where YOLOv4 has presented the highest accuracy [22]. Compared with the previous version, YOLOv4 features many significant improvements. YOLOv4 can be used as the optimal network for future object detection research and development [23].

Standard convolution operations have two disadvantages: The first is local feature extraction operations, which cannot directly extract global features from a more extensive range or image. In addition, the size of the convolution kernel is generally fixed (i.e., 3×3), and cannot adapt well to changes in the attitude and shape of objects. Second, when the number of feature channels increases, the number of parameters of the convolution kernel will also become large, increasing the amount of calculation [24]. In order to address the above problems, many new convolution methods have been proposed, such as dilated convolution, deformable convolution, group convolution, and depth-wise separable convolution. Dilated convolution involves spacing adjacent elements of the convolution kernel by a certain number of pixels, which can increase the receptive field, but with partial loss of information. Deformable convolution uses the backpropagation error to automatically adjust the shape of the convolution kernel, thus allowing for adaptive localization and accurate feature extraction for objects of different shapes and sizes, with the disadvantage that a small part of the features will be lost. Group convolution can be regarded as a sparse convolution connection method: each output channel is connected to only a certain group of input channels. In this way, the data between the channels are not circulated, which may cause global channel information to be lost. Depth-wise separable convolution divides the standard convolution into a depth-wise convolution and a pointwise convolution, which can effectively reduce the number of network parameters; this is considered the mainstream lightweight method. As the number of network layers has been typically deepening in such models, applying these lightweight methods to each layer can somewhat reduce the computation. To further lighten the network, it is vital to increase the local receptive field and reduce the number of layers of the network.

This work focuses on applying a model to resource-limited edge devices and the need for both speed and accuracy. Based on the above analysis of object detection networks and lightweight networks, we chose to design an improved garbage object detection model based on YOLOv4. The local receptive field is expanded by dilated–deformable convolution without increasing the number of parameters, and the features of different layers are fused by combining the residual structure. Furthermore, the number of network parameters is reduced by depth-wise separable convolution. Due to the expansion of the receptive field, it is not necessary for many layers to have the same receptive field, thus reducing the number of network layers and making the network lightweight.

## 3. Methodology

### 3.1. Data Set

**Figure 1:**
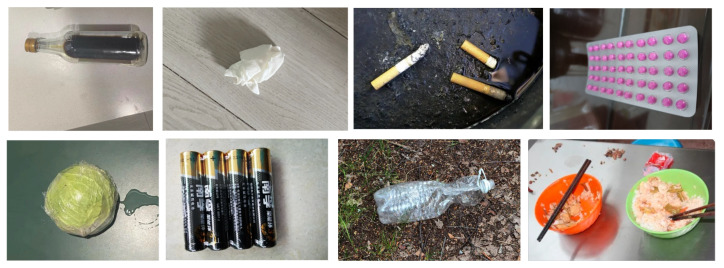
Sample self-built garbage dataset.

The TrashNet data set from Stanford University is currently the most used in garbage classification research, which consists of 2527 photos divided into 6 categories: plastic, metal, cardboard, paper, glass, and trash [25]. In China, garbage is divided into four main categories: recyclable garbage, harmful garbage, wet garbage, and dry garbage, with each category containing multiple types of waste. The TrashNet data set has few categories and does not match the categories of the Chinese garbage classification. In order to conform to the actual situation in China, we collected a dataset of domestic waste images according to the relevant standards. Some of the samples in the data set are shown in Figure 1, which are consistent with China’s garbage classification. The data set adopts the annotation format of VOC2007 and divides garbage into four categories and 11 sub-categories: recyclable garbage, food garbage, harmful garbage, and others. Recyclable garbage includes paper product, plastic container and glass container. Food garbage includes leftover, vegetable and fruit. Harmful garbage includes battery and drug. Other garbage includes soiled paper, chopstick and cigarette end. The total number of images was 4561, and each waste class was randomly divided such that 80% would be used for training, and the remaining 20% would be used for testing. The distribution of the number of images in each category in the training and test sets was the same.

### 3.2. Pre-Processing and Data Augmentation

**Figure 2:**
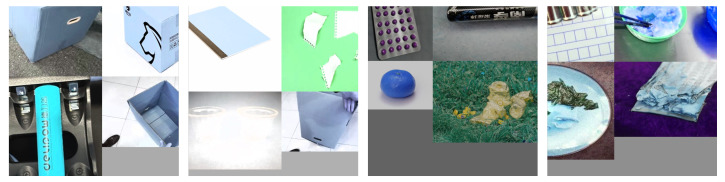
Improved data augmentation method for the garbage data sets.

To detect small objects in complex scenes, we improved the Mosaic data augmentation [26] method to improve the network performance. Mosaic data augmentation was proposed in YOLOv4, which is an improved version of CutMix [27]. The principle was to perform random scaling, cropping, stitching, and color gamut transformation of four images to obtain a new image while updating the object boxes in the image, as shown in Figure 2. To a certain extent, this increased the proportion of small objects in the data set, which was conducive to learning small object features by the model, reducing the training difficulty and training costs, and improving the training speed.

Pre-processing includes data enhancement and normalization. The data enhancement process involves image flipping, scaling, segmentation, stitching, as well as the box position update. Normalization involves dividing the enhanced data by 255.

### 3.3. Algorithm Design

#### 3.3.1. YOLOv4

You Only Look Once (YOLO) is a viral and widely used algorithm [28], which is known for its excellent object detection performance. In 2015, the first version of YOLO was introduced by Redmon et al. [29]. Over the past few years, scholars have published YOLOv2, YOLOv3, YOLOv4, and YOLOv5. The core of the YOLO object detection algorithm lies in the small size of the model and its fast computation speed. YOLOv3 introduced the feature pyramid network (FPN) [30] structure to obtain feature maps at three scales to improve the detection accuracy for multi-scale targets. YOLOv3 has a better base classification network, Darknet53, which uses a residual structure, and the number of layers is improved, compared to YOLOv2. YOLOv3 has three prior boxes at each position, and *k*-means is used to obtain the sizes of these prior boxes. YOLOv4 is a significant improvement over previous versions, with substantial improvements and very high performance. The backbone of YOLOv4 is CSPDarknet53, which adds a residual block to the original ResBlock, along with SPP, PAN, CBN, and other structures. YOLOv4 provides a faster and more accurate object detector than other object detection networks. Jiang et al. have validated many of the improvement points in YOLOv4, which they chose to use to improve its classification and detection accuracy, suggesting that YOLOv4 can be used as a best practice for future research and development. YOLOv5 provides a variety of network architectures for more flexible use and the model size is very lightweight and comparable to YOLOv4 in terms of accuracy; however, people still have reservations about YOLOv5, as it is not as innovative as YOLOv4 [23]. YOLOv4 has many improvements and performs very well; therefore, we chose to improve YOLOv4. The following briefly introduces the main features of YOLOv4.

**Figure 3:**
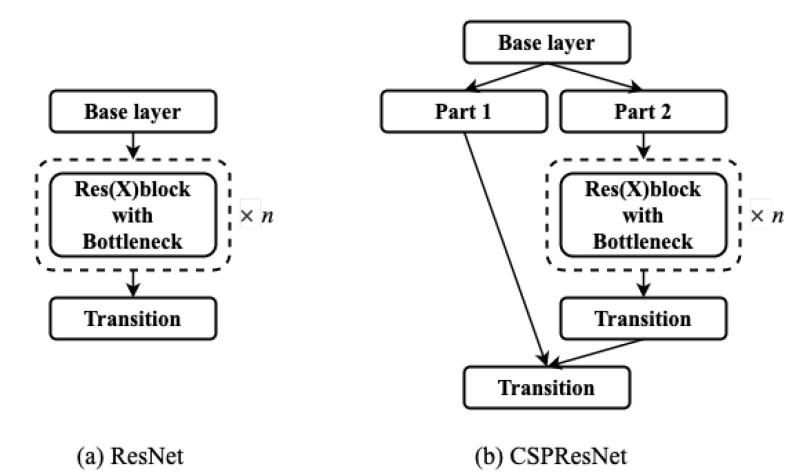
YOLOv3 and YOLOv4 feature fusion strategies: (**a**) ResNet for YOLOv3; (**b**) CSPResNet for YOLOv4.

CSPDarknet53 is the backbone feature extraction network of YOLOv4, which mainly borrows the idea of CSPNet [31] on Darknet53 and improves the residual network structure module. The specific structure is shown in Figure 3. CSPResNet can be seen as the original residual part, divided into two different paths for convolution separately. Afterward, the feature information is fused. CSPResNet retains the advantage of the feature reuse of ResBlock, but prevents excessive duplicate gradient information by truncating the gradient stream.

**Figure 4:**
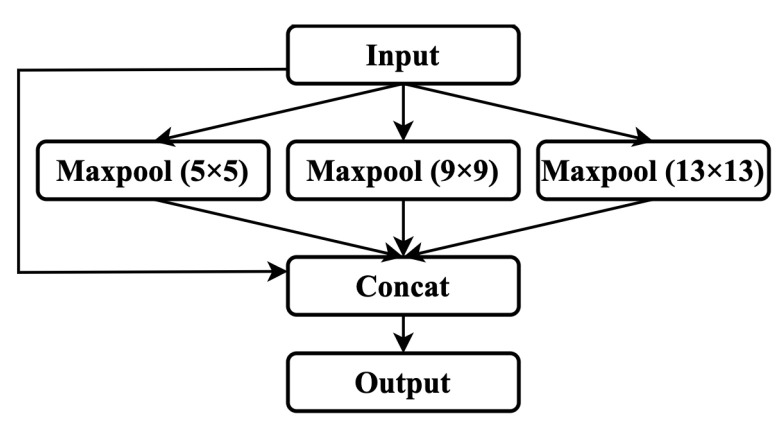
Network structure with a spatial pyramid pooling layer.

The spatial pyramid pooling (SPP) layer adopts the maximum pooling method with kernels of 1×1, 5×5, 9×9, and 13×13, and then splices the pooled feature maps to achieve the fusion of feature maps at different scales. SPP can effectively increase the extraction range of the backbone features, significantly separate the essential contextual features, and it hardly affects the network’s speed. The structure of SPP is shown in Figure 4.

**Figure 5:**
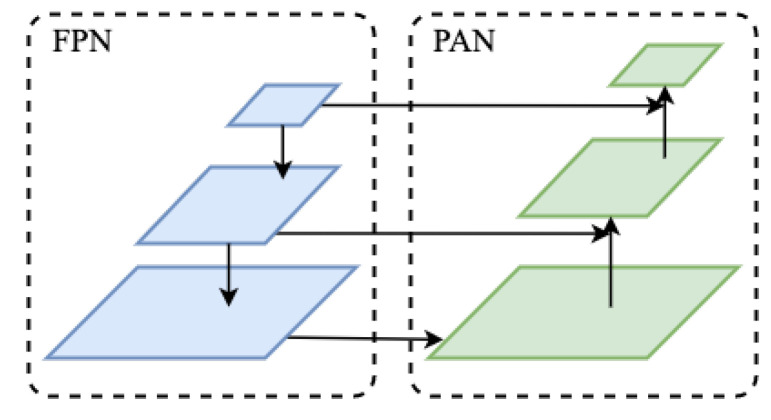
FPN and PAN for YOLOv4.

The feature pyramid network (FPN) conveys features in a top-down manner, while the path aggregation network (PAN) draws on the innovation of PANet in image segmentation, and conveys features from the bottom-up. FPN and PAN are combined to aggregate parameters from different backbone layers to different detection layers, in order to accelerate the fusion of features at different scales and further improve feature extraction. The FPN and PAN structures are shown in Figure 5.

For the loss, we adopted CIoU loss, instead of box position loss. CIoU adds two penalty terms, compared to IoU, considering the three geometric elements of overlap area, centroid distance, and aspect ratio [32]:

(1) $$R_{CIoU}=\frac{\rho^{2}\left(b,b^{gt}\right)}{c^{2}}+\alpha v,$$

where $\rho^{2}\left(b,b^{gt}\right)$ is the Euclidean distance between the prediction box and the center point of the truth box, *c* is the diagonal distance of the smallest closed region that can contain both the prediction box and the truth box, α is the weight function, and *v* is used to measure the consistency of the aspect ratio.

(2) $$\alpha =\frac{v}{(1-IoU)+v},$$

(3) $$v=\frac{4}{\pi^{2}}{\left(arctan\frac{w^{gt}}{h^{gt}}-arctan\frac{w}{h}\right)}^{2},$$

(4) $$L_{CIoU}=1-IoU+\frac{\rho^{2}\left(b,b^{gt}\right)}{c^{2}}+\alpha v,$$

(5) $$loss=L_{CIoU}+L_{conf}+L_{cls},$$

where $L_{con\;f}$ is the confidence prediction loss and $L_{cls}$ is the class prediction loss.

#### 3.3.2. Focus

**Figure 6:**
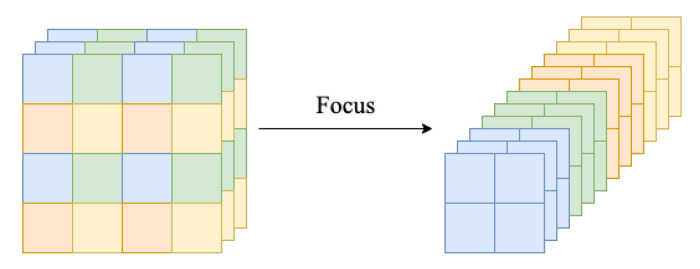
Structure of Focus.

‘Focus’ was proposed by Glenn Jocher et al. [33], which consists of a slice operation on the image before it enters the backbone network. The specific operation is to take a value for every pixel in an image, similar to neighborhood downsampling, in order to obtain four feature layers with the same width and height as the channel. Stacking these four feature layers expands the input channel by a factor of four without losing the information of the original input. The Focus structure is shown in Figure 6. After the Focus module, the channel was changed to 12, which was expanded to 64 by one convolution. Using the Focus module and one convolution, instead of two convolutions, can effectively reduce the number of parameters and computation, thus increasing the speed of forward- and backpropagation.

#### 3.3.3. DCSPResNet

**Figure 7:**
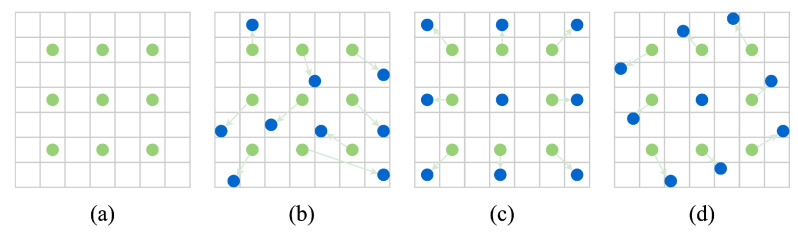
Illustration of the sampling locations in 3×3 standard and dilated–deformable convolutions. (**a**) Regular sampling grid (green dots) for standard convolution. (**b**) Dilated–deformable sampling locations (dark blue dots) with enhanced offset (light blue arrows) in the dilated–deformable convolution. (**c**,**d**) Particular cases of (**b**), showing that the dilated–deformable convolution generalizes the various transformations of the scale, (anisotropic) aspect ratio, and rotation [34]. The dilated–deformable improves on the deformable convolution.

Standard convolution kernels are regular shapes, such as rectangles or squares; however, convolution kernels with regular shapes often limit the effectiveness of feature extraction. The convolution kernel in deformable convolution can have any shape and can be automatically shaped to fit the critical region of interest of the network model, based on the backpropagation error of the network. Deformable convolution can better extract features accurately and improve the detection accuracy. The dilated–deformable convolution is composed of dilated convolution [9] and deformable convolution [10], thus reducing the grid effect of dilated convolution. It can enhance the local receptive field and accurately extract features. Its structure is shown in Figure 7. The dilated–deformable convolution learns offset and mask through an additional convolution layer. Figure 7b–d show the convolution process after adding the offset, so the parameters of deformation are generated adaptively by the input feature layer. 

**Figure 8:**
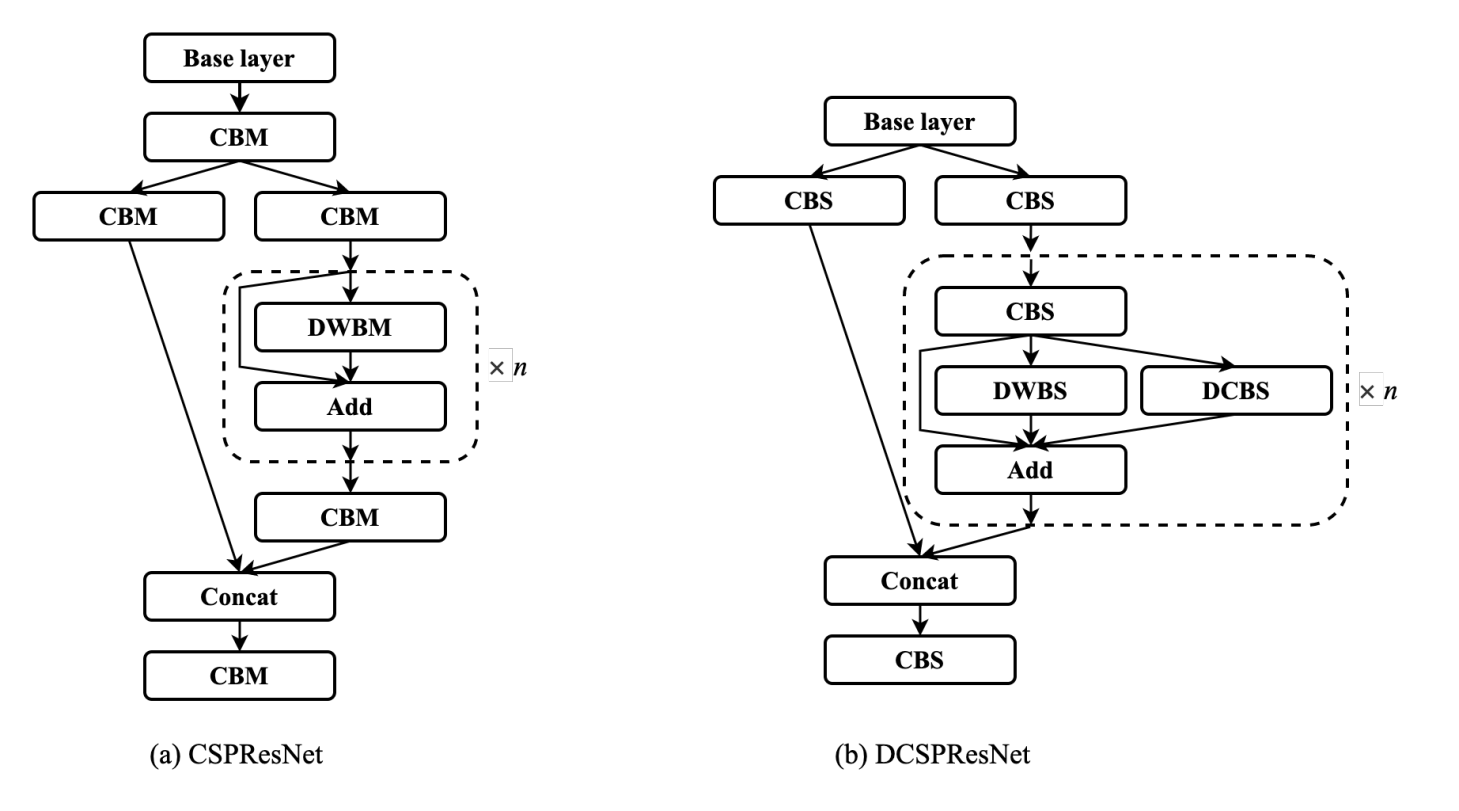
Network structure of CSPResNet and DCSPResNet. CBM consists of convolution, batch normalization, and the Mish activation function. DWBM consists of depth-wise separable convolution, batch normalization, and the Mish activation function. The CBS, DWBS, and DCBS structures are shown in Figure 9.

The convolution kernel sizes used in the standard YOLOv4 model for convolution operations are 1×1 and 3×3, which are on the small side. The use of smaller convolutional kernels in the model can somewhat reduce the number of parameters. The size of the convolution kernel corresponds to the size of the receptive field, and a smaller convolution kernel has a smaller local receptive field. In order to increase the receptive field, the number of network layers must be deepened. Using a large-sized convolution kernel can increase the local receptive field and extract more features, but will increase the number of network parameters. The above problem can be solved effectively by the use of dilated–deformable convolution, but a small amount of information is inevitably lost. Sanjeev Arora et al. have shown that, if a convolutional neural network is considered as a probability distribution of a data set, then clustering the outputs with high correlation can build an optimal network structure [35]. The dilated–deformable convolution is used as a residual block of the residual structure. The clustering of these nodes with high correlation is connected, equivalent to simultaneous convolution at multiple scales to build a structure with multiple branches, as per the above theory. CSPResNet has a residual structure, based on which the dilated–deformable convolution is used as a new block of the residual structure. A convolution block is added to extract features before the residual structure. The first and last layers of CSPResNet usually contain convolution blocks; thus, the first convolution block of CSPResNet and the last convolution block of the residual structure were removed. The specific structure is shown in Figure 8.

#### 3.3.4. YOLOG

The network structure in YOLOv4 was designed with multiple repetitive convolution blocks, in order to extract the features thoroughly, where the computational burdens of these repetitive convolution blocks were very high throughout the whole network. Therefore, these repeated convolution blocks are key to the lightweight nature of the network.

CSPDarknet extracts features through five CSPResNet modules, with cycles of 1, 2, 8, 8, and 4, respectively, for a total convolution count of 72. The number of convolution layers, computations, and parameters of CSPDarknet are very high, as shown in Table 1, making it unfavorable for operation in resource-limited embedded devices. Therefore, we replaced the first CSPResNet module in CSPDarknet with a Focus module, thus reducing four convolution layers. The remaining four CSPResNet modules were replaced with three DCSPResNet modules, with the number of cycles of these three modules set to 1, 2, and 2, and the dilation parameters set to 1, 2, and 4, respectively. The partial convolution was replaced with a depth-wise separable convolution. The total number of convolutional layers of the improved backbone feature extraction network was 36, which is half the number of convolutional layers before the improvement, with 27 times fewer computations and 87 times fewer parameters.

SPP consists of convolution and pooling parts, where the convolution part consists of three consecutive convolution blocks. The convolutional part was replaced by a depth-wise separable convolution block and a DCSPResNet module, placed before and after the pooling operations, respectively. FPN and PAN consist of downsampling, upsampling, and four convolutional parts, where the convolutional part consists of five consecutive convolutional blocks. Downsampling and DCSPResNet modules were used to replace the convolutional part. The YOLO head consists of a convolution block and a basic convolution. We removed the convolution block and kept only the basic convolution.

All activation functions in the network use the SiLU activation function, which is one of the Swish functions. SiLU has upper bound-free, smooth, and non-monotonic properties. Vasu Singla et al. [36] demonstrated that SiLU has a small generalization gap between training and prediction, and does not present the double descent phenomenon.

**Figure 9:**
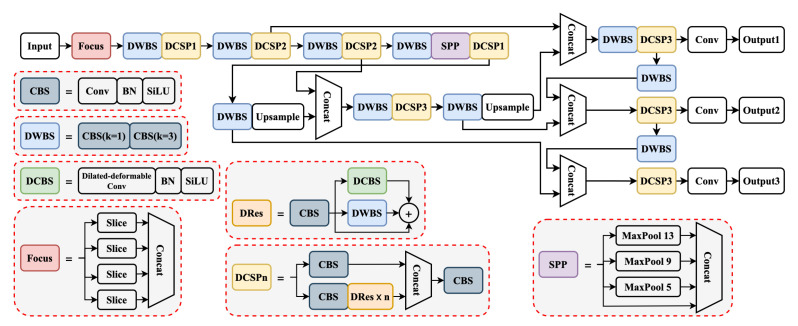
The architecture of YOLOG. The red-dashed boxes show the structures of each sub-module.

The final structure is shown in Figure 9. The original model had 59.65 Gflops of computation and 63.99 M parameters, while the improved model had 6.05 Gflops of computation and 6.17 M parameters.

### 3.4. Performance Indices of the Object Detection Model

Object detection is usually evaluated using the precision (*P*), recall (*R*), precision–recall (PR) curve, F1 Score (F1), average precision (AP), mean average precision (mAP), and frames per second (FPS), in order to assess model performance. The first two are calculated as shown in Equations (6) and (7):

(6) $$P=\frac{TP}{TP+FP},$$

(7) $$R=\frac{TP}{TP+FN},$$

where TP, TN, and FN are the numbers of true positives, true negatives, and false negatives, respectively. The precision and recall should be as high as possible.

The PR curve can intuitively show the performance of the classifier. It is a curve formed based on a certain threshold with recall as the abscissa and precision as the ordinate. The closer the PR curve is to the upper right, the better the network performance.

The F1 score is an important measure of the classification task, which is a summed average of precision and recall. The F1 score can be calculated using Equation (9). F1 combines the results of *P* and *R*. When F1 is high then it can indicate that the test method is more effective.

(8) $$F1_{k}=2*\frac{P_{k}*R_{k}}{P_{k}+R_{k}},$$

(9) $$F1={\left(\frac{1}{n}\sum_{k=1}^{NC}F1_{k}\right)}^{2},$$

AP is the most common object detection performance index [37], which is defined as the area under the PR curve, calculated as follows:

(10) $$AP=\int_{0}^{1}P\left(r\right)dr,\;$$

where P(r) denotes the precision as a function of the recall (*r*).

The mean average precision (mAP) is equal to the mean of the AP sums for all detection classes, which are used to evaluate the average measurement accuracy in multiclass object detection. Thus, it is often considered the ‘overall performance’ of the detection model [38]. The formula for calculating the mAP is as follows:

(11) $$mAP=\sum_{k=1}^{NC}\frac{AP_{k}}{NC},\;$$

where $AP_{k}$ is the AP of the kth object class, and NC refers to the number of object classes.

In the evaluation metrics of the COCO data set, AP, $AP_{0.5}$, and $AP_{0.75}$ are typically used to evaluate the object detection performance [39]. Here, the AP is essentially the mAP; where the AP is averaged over multiple intersection over union (IoU) values. Specifically, we used 10 IoU thresholds from 0.50 to 0.95 in steps of 0.05. Moreover, $AP_{0.5}$ is the mAP with an IoU threshold of 0.5; while $AP_{0.75}$ is the mAP with an IoU threshold of 0.75. AP, $AP_{0.5}$, and $AP_{0.75}$ are used as the evaluation metrics of the model in the subsequent results.

FPS is the number of frames per second transmitted, it indicates the number of images detected per second. It is considered real-time when the FPS is greater than 30.

The floating point operations (flops) are measures of the model complexities. Giga floating-point operations (Gflops) are used in the paper to represent the computation of the model at the 32-bit floating point.

### 3.5. Training Strategies and Experimental Setup

**Figure 10:**
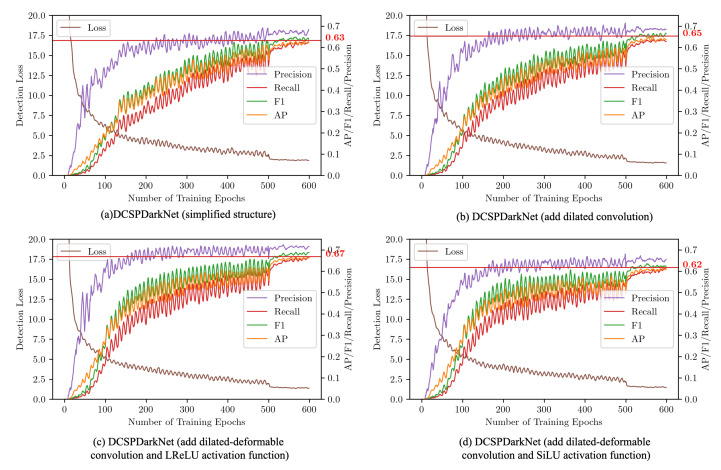
The training curve in the ablation experiment on the garbage data set: (**a**) the simplified model of the network; (**b**) the model after adding dilation convolution to (**a**); (**c**) the model after adding deformation convolution and the LReLU activation function to (**a**); and (**d**) the model after adding deformation convolution and the SiLU activation function to (**a**).

As the network was heavily modified, there was no suitable pre-trained model for transfer learning and, thus, we had to train the model from scratch. There were many hyperparameters to be tuned in the model training process, and we focused on the four aspects of the optimizer, loss, learning rate scheduler, and data augmentation. The optimizer uses Adam. Adam is invariant to diagonal rescaling of the gradients, and is well suited for problems that are large in terms of data. Moreover, the hyperparameters have intuitive interpretations and typically require little tuning. To prevent overfitting, we set the weight decay to 0.0005. The location loss was calculated using CIOU Loss. CIoU added two penalty terms, compared to IoU, considering the three geometric elements of overlap area, centroid distance, and aspect ratio. The learning rate uses the cosine annealing algorithm. Cosine annealing periodically increases and decreases the learning rate following a cosine function. Compared to the traditional method, the cosine annealing function will attempt to explore different local minima and expand the search space of the objective landscape. We used data augmentation to improve the performance of the model, and we found that data augmentation is not more beneficial for training in the later stages, so we turned off data augmentation in the later stages. In our experiments, we divided the network training into two stages: data augmentation were on in the first stage, and data augmentation were off in the second stage. The specific configurations are shown in Table 2. We iterated 500 iterations in the first stage and 100 iterations in the second stage, as presented in Figure 10.

All models were trained using a computer with two Intel Xeon Silver 4210R 2.40 GHz, 128 GB RAM, and two NVIDIA GeForce RTX 3090; speed tests were performed on a Jetson AGX Xavier.

## 4. Results

### 4.1. Results of the Ablation Experiment

In order to demonstrate the contribution of each improvement point to the performance improvement of the network, the improvement points proposed in this study were introduced into the network separately, and an ablation experiment was performed. The ablation experiment included the introduction of the cropped structure, dilated convolution, dilated–deformable convolution, and activation function. The training procedure for the ablation experiment on the garbage classification data set is shown in Figure 10. The figure indicates that dilation and deformation convolution accelerated the model convergence speed and accuracy. Comparisons of the values of the final trained results for each model are shown in Table 3. From the table, we can see that YOLOv4 had good performance and could achieve a high accuracy rate, but with the drawback that the number of model parameters and computations were too large. After adjusting the network structure, the number of parameters and the number of computations were reduced to one-tenth of that of the baseline model. However, there was still a gap between the AP with the baseline model. The dilation module was added to the restructured model to enhance the local receptive fields and improve the network performance. After adding dilated–deformable convolution, the model computation, parameters, and AP were slightly improved. Based on this, the dilated convolution was replaced by dilated–deformable convolution (i.e., the DCSPResNet module in the paper) in order to reduce the parameters and computations further while enhancing the local receptive field; in this case, the AP was improved by 2.04%. We also compared the performance of the FReLU activation function in the network, by replacing the activation function from SiLU to FReLU. The parameters of the FReLU activation function were calculated by convolution, which increased its number of parameters and computation. However, its accuracy was not as good as that of the former SiLU activation function, as the accuracy decreased by 5.2%. Finally, using DCSPResNet, the SiLU activation function, and data augmentation, YOLOG achieved 66.7% AP in the household garbage data set, presenting a 1.85% improvement in accuracy, compared to the baseline network, while reducing the number of parameters and computations to one-tenth of the baseline model, thus achieving a higher prediction accuracy while maintaining lightweight properties.

### 4.2. Comparison of Recognition Performance

There are various object detection models based on convolutional neural networks. In order to illustrate the performance of YOLOG, experimental comparisons were performed against a variety of state-of-the-art mainstream models. The YOLOv3, YOLOv4-tiny, and YOLOv5-s models were selected for the experiments, which were trained uniformly using the garbage classification data set in this paper. All models should have been retrained, as with YOLOG; however, we found that these models were slow to converge and did not achieve high accuracy when we started training again. Therefore, initializations of their weights were performed using transfer learning. The training process was also divided into two phases. In the first phase, the weights were initialized through transfer learning and data augmentation was used to conduct training 500 times; in the second phase, data augmentation was turned off and training was carried out 100 times. During the training process, the training losses of the models and the accuracies of the test sets were recorded after each training cycle was completed, in order to observe the model training and to ensure that each model converged when it finished training. Finally, the model weights were quantified to 16-bit widths by TensorRT and tested for speed on a Jetson AGX Xavier. The performance of each network is shown in Table 4. As TensorRT does not support dilated–deformable convolution, it was not possible to test the proposed model’s speed on the Jetson AGX Xavier, and we replaced the dilated–deformable convolution with dilated convolution for the test; therefore, as dilated–deformable convolution is faster, the model’s speed was theoretically underestimated in the table. As can be seen from Table 4, under the same conditions, AP, $AP_{0.75}$, and FPS than YOLOv3, YOLOv4, YOLOv4-tiny, and YOLOv5-S in the garbage detection task, and had the smallest Gflops; $AP_{0.5}$ was close to YOLOv5-S. YOLOG achieved excellent results in the garbage detection tasks, proving the performance advantages of YOLOG. YOLOG is improved based on YOLOv4. The AP, $AP_{0.5}$, and $AP_{0.75}$ of YOLOG were 1.85%, 0.7%, and 1.7% higher than YOLOv4, respectively, as well as the number of parameters, Computations of the model are only 1/10 of YOLOv4.

The experimental results indicate that the overall accuracy of the garbage object detection network based on accurate local receptive field expansion proposed in this paper is significantly improved, with respect to the garbage image object detection problem, compared with the considered mainstream methods. Therefore, YOLOG is a very effective model for garbage image object detection.

### 4.3. Performance on the Public Dataset

In order to further examine the advantages of YOLOG and the generality of transform to other tasks, we set up a comparative experiment to compare the performance of YOLOG and other CNN models on the public datasets PASCAL VOC [40]. The dataset includes PASCAL VOC2007 and PASCAL VOC2012 with 20 target classes, 16,551 training images, and 4952 test images. The experimental results on the PASCAL VOC dataset are shown in Table 5. The AP and $AP_{0.5}$ of YOLOG are 4.33% and 8.3% lower than YOLOv4, respectively, and the AP and $AP_{0.5}$ are 2.64% and 0.66% higher than YOLOv5s, respectively; the computation is smaller than both YOLOv4 and YOLOv5s. YOLOv4-tiny is the least computationally intensive, but AP and $AP_{0.5}$ are lower. Therefore, the method still has good detection capability in the public object detection dataset. Thus, the generality of the proposed method is verified, and the model can be adapted to different datasets or scenarios by retraining the proposed model structure.

### 4.4. Object Detection Test

**Figure 11:**
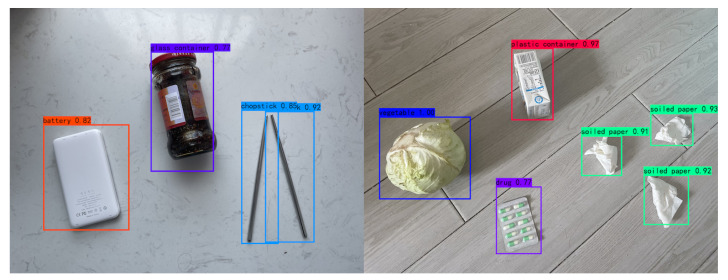
Detection results of garbage pictures.

We took a set of images (of common household garbage) to test YOLOG and passed these images to the deployed YOLOG model. We boxed out the detected objects and marked the classes and confidences on the top left of the box. The results are shown in Figure 11. It can be seen that the model has a strong ability to generalize and can accurately identify garbage.

## 5. Conclusions

Properly sorting garbage and improving recycling rates serve to protect the environment and conserve resources. Current sorting methods rely too much on manual intervention and are susceptible to factors, such as personal bias, attention, and responsibility. The separation of hazardous waste can also affect the health of workers. Efficient and reliable automatic garbage classification technology is an inevitable trend in social development, and applying artificial intelligence techniques to garbage classification can improve efficiency and reduce labor costs. Therefore, in this paper, a lightweight and efficient garbage object detection model, YOLOG, was designed. YOLOG is an improved model based on YOLOv4 with optimized data augmentation, CSPResNet, overall structure, and a new activation function. The contributions of this paper are as follows:

1. We improved the CSPResNet structure using dilated–deformable convolution to accurately expand the receptive field and extract features more effectively.
2. A lightweight garbage object detection network, named YOLOG, was designed to ensure real-time and accurate results. It simplifies the hardware requirements, reduces computational costs, and meets the needs of practical applications.
3. We presented comparative experiments with other advanced networks on garbage and public datasets, respectively, to demonstrate the effectiveness of YOLOG.
4. YOLOG allows for the efficient detection and classification of all types of domestic garbage using edge devices (e.g., Jetson AGX Xavier).

YOLOG can be applied in intelligent garbage sorting devices, such as garbage sorting machines, garbage cans, and refuse collection points. YOLOG has a lightweight structure with high accuracy, and can operate in real-time in resource-limited edge devices. The method proposed in this paper has great academic significance and practical application value for applying artificial intelligence technology in garbage classification.

This paper still had some limitations, which could be improved in future work. First, there were various types of garbage that the data set used and could not fully cover, and the garbage heap scenario was not considered. Moreover, in the experiments, we found that for object occlusion and relatively rare object recognition, the YOLOG recognition effect was poor. Second, the optimized network in this paper can operate efficiently in real-time on edge devices, such as Jetson, but cannot do so in real-time on cheaper, lowerperformance CPU devices. In future work, we intend to study the detection scheme with target occlusion, expand the garbage data set, optimize the network for CPU hardware, and use deep learning acceleration libraries, such as MKL-DNN, to achieve efficient garbage object detection at the CPU. Using these improvements in a generic object detection scheme is also possible. Our ultimate goal is to apply YOLOG to garbage classification facilities, in order to effectively promote resource recycling and sustainable social development.

## Figures and Tables

**Table 1:** Comparison of backbone network structures.

Backbone	Gflops	Params (M)
CSPDarknet	34.49	26.617
DCSPDarknet	1.255	0.304

**Table 2:** Strategy for two-stage model training.

Epoch	Strategy	Method
1–500	Optimizer	Adam (weight decay = 0.0005)
	Loss	CIoU
	Learning rate scheduler	CosineAnnealingLR
	Data augmentation	Mosaic
500–600	Optimizer	Adam (weight decay = 0.0005)
	Loss	CIoU
	Learning rate scheduler	CosineAnnealingLR
	Data augmentation	None

**Table 3:** Roadmap of YOLOG, in terms of AP(%), on the garbage data validation set. All models were tested at 416×416 resolution.

Method	AP(%)	Gflops	Params (M)
YOLOv4 baseline	64.85	59.65	63.99
+ restructuring + SiLU	62.95 (−1.9)	5.55 (−54.1 )	5.86 (−58.13)
+ dilated conv	64.66 (+1.71)	8.42 (+2.87)	8.81 (+2.95)
+ dilated–deformable conv	66.7 (+2.04)	6.05 (−2.37)	6.17 (−2.64)
+ FReLU	61.50 ( −5.2)	8.45 (+2.4)	8.81 (+2.64)

Green fonts indicate performance improvements.

**Table 4:** Comparison of the speed and accuracy of different object detectors on the garbage data set. The FPS values in this table were measured without post-processing in the Jetson NV AGX Xavier.

Method	Backbone	Size	FPS	AP(%)	${\mathrm{AP}}_{0.5}$	${\mathrm{AP}}_{0.75}$	Gflops	Params (M)
YOLOv3	Darknet-53	416	46.58	64.53	94.22	73.41	65.42	61.58
YOLOv4	CSPDarknet-53	416	41.45	64.85	93.88	73.11	59.65	63.99
YOLOv4-tiny	CSPDarknet-53	416	230.9	37.97	76.23	32.12	6.84	5.9
YOLOv5-S	Modified CSP v5	416	125.2	64.47	95.07	73.84	6.93	7.09
YOLOG(ours)	DCSPDarknet	416	127.0 ^1^	66.7	94.58	75.11	6.05	6.17

^1^ We replaced dilated–deformable convolution with dilated convolution. Theoretically, dilated–deformable convolution is a little faster than dilated convolution.

**Table 5:** The results of different models on the PASCAL VOC 07+12.

Method	Size	AP(%)	${\mathrm{AP}}_{0.5}$	Gflops
YOLOv4	416	60.07	88.26	59.71
YOLOv4-tiny	416	42.43	79.07	3.43
YOLOv5-S	416	53.10	79.30	6.98
YOLOG(ours)	416	55.74	79.96	6.08

## Data Availability

Not applicable.
